# The Influence of Antiobesity Media Content on Intention to Eat Healthily and Exercise: A Test of the Ordered Protection Motivation Theory

**DOI:** 10.1155/2014/954784

**Published:** 2014-11-19

**Authors:** Raeann Ritland, Lulu Rodriguez

**Affiliations:** ^1^Department of English, Iowa State University, 317 Carver Hall, Ames, IA 50011-2060, USA; ^2^Agricultural Communications Program, University of Illinois at Urbana-Champaign, 274 Bevier Hall, 905 South Goodwin Avenue, Urbana, IL 61801, USA

## Abstract

This study extended the ordered protection motivation framework to determine whether exposure and attention to antiobesity media content increases people's appraisals of threat and their ability to cope with it. It also assesses whether these cognitive processes, in turn, affected people's intention to abide by the practices recommended to prevent obesity. The results of a national online survey using a nonprobability sample indicate that attention to mediated obesity and related information significantly increased people's intention to exercise as well as their overall coping appraisals (the perceived effectiveness of the recommended behaviors and their ability to perform them). Likewise, increased threat and coping appraisals were both found to significantly influence people's intention to exercise and diet. Coping (rather than threat) appraisals more strongly predicted behavioral intent. Following the attitude-behavior literature, behavioral intention was used as the most proximate predictor of actual behavior (i.e., stronger intentions increase the likelihood of behavior change).

## 1. Introduction and Problem Statement 

As a medical condition, obesity has grown into an epidemic that has overwhelmed the American public. From 1991 to 1998, the rate of obesity (defined as people with a body mass index or BMI of over 30) rose from 12% to 17.9% [1]. The steady increase in the incidence of obesity across all states and across demographic groups grew to 18.9% in 1999 with 16 states registering obesity rates above 20% [2]. By 2004, Wang and Beydoun [3] report that obesity rates had increased to 32%. By 2012, the Journal of the American Medical Association, reports that some 34.9% of adults above the age of 20 were obese [4]. Although not a dramatic increase, the incidence remains high, necessitating surveillance. Obesity rates among ethnic groups vary. Non-Hispanic black adults show the highest rate, 47.8%, followed by Hispanics at 42.5%, and non-Hispanic whites at 32.6%. Non-Hispanic Asians drew the lowest rate at 10.8% [4].

The figures worsen when those who are overweight (defined as people with a BMI of over 25) are included—66% of American adults were found to be overweight or obese in 2007, a figure that is predicted to climb to 75% by 2015. Of this number, 41% will be obese [3]. More alarmingly, based on national survey data collected between 1970 and 2004, Wang et al. [5] project that by 2030, 86.3% of American adults will be overweight or obese; 51.1% will be specifically obese. Figures from Ogden et al. [4] show a slight increase since 2007, with 68.5% considered to be overweight or obese.

The trend is disconcerting considering that the complications associated with the condition are many, resulting in health and financial burdens to individuals and to society at large. Health problems include an array of chronic diseases such as cardiovascular ailments, diabetes [1], hypertension, asthma, angina, lung disease [6], certain cancers [7, 8], and premature death [9]. These complications are likely to tax the health financial system. Sturm [6] found obesity to be “associated with a 36% increase in inpatient and outpatient spending and a 77% increase in [the cost of] medications” (page 245). Wang et al. [5] project that “total health care costs attributable to obesity and [being] overweight will more than double every decade. By 2030, health care costs…could range from $860 to $956 billion, which would account for 15.8%–17.6% of total health care costs, or for one in every six dollars spent on health care” (page 2,329).

Experts recommend a combination of healthy diet and exercise to fight obesity. However, as the CDC [10, 11] reports, just 32.5% of adults consume fruits two or more times per day, and only 26.3% eat vegetables more than three times a day. More recent figures for the state of Iowa show that just 27.5% of adults follow the recommended daily level of fruit intake (two or more per day) and just 21.9% abide by the suggested level of vegetables in the diet on a daily basis [12]. About 44% of Iowa adults perform at least 300 minutes of moderate-intensity or 150 minutes of vigorous-intensity aerobic activity per week [12]. In fact, 24.2% report no physical activity within the last month. Thus, people are not meeting the recommended daily intake of fruits and vegetables and the suggested physical activity levels.

As one-on-one conversations with medical practitioners become infrequent, the mass media (traditional and online) increasingly become the public's primary sources of information about health issues. Despite the availability of information to combat the condition, the obesity rate continues to climb. Do people find health-related information in the mass media useful? Do they use it to inform their decisions to adopt healthy behaviors? This study investigates the link between exposure and attention to mass media antiobesity content, the cognitive processes people go through to assess the threat and the proposed solutions, and their intention to practice the behaviors recommended to solve the problem.

## 2. Literature Review and Theoretical Framework

### 2.1. Sources of Health Information

When it comes to health, Americans generally rely on mediated sources. A survey of 452 young adults indicated they learn about health mostly from television, followed by radio, print, the Internet, and social networking sites [13]. Of these channels, media and information service companies (e.g., Dr. Oz, The Doctors, iVillage, and WebMD) make up 56% of the sources people use, while government organizations (e.g., the CDC and the FDA) and companies that produce health products make up 16% each [14].

A year later, Colby et al. [15], surveying a random sample of 437 adults, reported that people get information about health and health-related topics from the Internet (28.3%), newspapers (26.4%), and postal service materials (22.3%). Generally, older people depend on newspapers; younger ones prefer online sources.

Beaudoin and Hong [16] analyzed data from a national telephone survey of 700 adult Americans conducted in 2007 and observed a dramatic rise in health information seeking via the Internet—62% reported having sought information online, a habit reported by youngsters, women, and those with higher education. Television also figured prominently as a health information source, with 64% reporting they get health information primarily from TV. Nonwhites were the most common users of this medium. Newspapers were the choice of older, highly educated, and nonwhite individuals.

Of the available media channels, television and its effects on unhealthy eating habits has been the subject of considerable debate. The Council on Communications and Media [17] cites multiple studies linking television viewing to obesity, but the findings are mixed. Some report a decline in physical activity (including exercising) with increased TV viewing; others did not [17]. Beaudoin and Hong [16] found no effect of mass media exposure on physical activity but reported that media exposure did significantly predict fruit and vegetable intake, unhealthy snacking, and soft drink consumption. They also noted that newspaper reading is strongly associated with increased intake of fruits, vegetables, and unhealthy snacks. Soft drink consumption rose with television viewing, but the relationship was not causal. Moreover, information seeking via the Internet did not correlate with performing more physical activity and healthier eating habits [16].

### 2.2. Obesity in the Media

The findings of these foregoing studies prompted researchers to examine how obesity and obese people have been portrayed in the mass media. Medical surveys reveal that the problem is more acute in developed countries. At least half of the population in 13 high-income countries has been reported to be overweight or obese [18]. Experts say this phenomenon can be attributed to both personal and social factors [19]. An analysis of mass media depiction of obesity conducted by Kim and Willis [20] indicates that before 2004, obesity was blamed on personality traits (e.g., poor eating habits), lack of exercise, and modern (sedentary) lifestyles. After 2004, however, the problem began to be attributed to social factors, such as the habits of peer groups, family eating patterns, and other influences in a person's social environment. Barry et al. [19] found evidence for both personal- and industry-level causes of childhood obesity in national and regional news media outlets. Heuer et al. [21] analyzed five major news websites and discovered that victim-blaming is still very much alive.

What is the nature of media portrayal? Heuer et al. [21] found that media depictions of obese individuals were more likely to be negative. Of the 549 news photographs they examined, 72% had negative or stigmatizing portrayals of obese persons. Such depictions, the authors argue, are “not beneficial for motivating weight loss. Weight stigma is counterproductive for public health and increases the likelihood of [performing] unhealthy eating behaviors, avoidance of physical activity, impaired weight loss efforts, and decreased use of preventive health services” (page 975).

Ries et al. [22] agree that media portrayals can shape people's understanding of obesity and the actions people take to alleviate the threat. They can also influence people's notions of obese individuals as well as their attitudes toward obesity as a health condition. Following an experiment, the authors report that participants who saw negatively framed photographs registered more negative attitudes toward obese people than those who saw pictures with positive frames. In addition, participants thought that obesity results mainly from overeating and that being overweight does not constitute a health problem.

Studies have shown mixed results regarding the influence of exposure and attention to antiobesity media content on the comprehension of health messages. Tian and Robinson [23], doing a secondary analysis of the Health Information National Trends Survey II data, conclude that incidental exposure to online information is positively associated with Internet use, active health information seeking, and the use of health information gleaned from the traditional media. Exposure also positively correlated with health knowledge. In short, greater media exposure translates to more knowledge about health issues. Likewise, Shapiro et al. [24] and Shapiro [25] found that exposure to advertisements influences cognitive processes that lead to buying behavior even without the explicit memory of exposure. These findings provide evidence that exposure to information affects knowledge and subsequent behavior.

Researchers also document that attention to mediated health content is a significant predictor of knowledge gain [23, 26, 27] and preventive behaviors [28, 29].

Given the foregoing literature, this study asks the following question.

RQ1: what channels do people refer to for obesity and related media content? To what extent are they exposed to and how much attention do they pay to this type of media content?

### 2.3. The Ordered Protection Motivation Model

Getting people to eat healthily and to exercise is a daunting task because “changing people's food habits can be an intervention into their culture, society, and relationships” [30, page 466]. How do people decide to abide by the general recommendations about how to prevent obesity? Several theoretical models have been proposed to explain the mechanisms of behavioral change. Of these, Rogers' [31] protection motivation theory (PMT) stands out as having considerable explanatory power. It posits that the higher the perception of threat is, the more likely people are motivated to change their behavior as long as the response or coping strategy is seen as effective in reducing that threat. In his original proposition, Rogers [31] argues that people respond to persuasive communication via cognitive processes that fall into two categories: threat appraisal and coping appraisal. Perceived severity, one's perception of how serious the consequences of the health problem are, and perceived vulnerability, a person's perception of the likelihood of contracting or developing the condition, combine to form a person's threat appraisal. Response efficacy and self-efficacy make up a person's coping appraisal of the situation. Response efficacy is the perceived effectiveness of the suggested action or behavior in treating or combating the problem. Self-efficacy is a person's belief in his/her ability to perform the recommended action.

**Figure 1:**
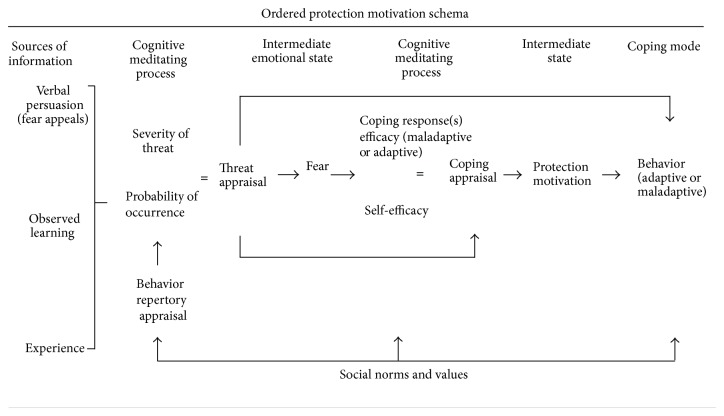


In their ordered protection motivation model (OPM), Tanner et al. [32] sought to improve PMT in four ways: (1) by emphasizing the emotions involved in the process, (2) by suggesting that appraisal mechanisms are more likely to occur sequentially, (3) by elucidating maladaptive coping behaviors, and (4) by introducing the social context of danger into the model because adaptive behaviors are influenced by norms. The OPM conceptual framework is shown in Figure 1.

Tanner et al. [32] proposed a linear sequence in which fear appeals are assumed to trigger emotional responses, which encourages engagement or cognitive processes that ultimately influence behavior. Thus, although fear may not actually cause behavior change, audiences are still likely to process fear appeals or threat-related information with the potential to lead to adaptive behavior. In addition, threat appraisals must lead to fear in order for coping appraisals to occur.

In their reformulation of the PMT, Tanner et al. [32] also introduced the notion of “maladaptive coping behaviors.” They explain that although coping behaviors seek to lessen threat and/or fear, people can behave in ways that reduce fear, but without diminishing the threat. An example is the propensity of sexually active college students to choose what they think are STD-free partners based solely on past success [33]. OPM also recognizes that social influences and pressures can thwart potential coping responses. For example, some may hesitate to use condoms because doing so implies that a partner has the infection. Thus, OPM highlights the importance of social norms in people's decision-making process.

Both models have been tested using experimental designs that involve splitting participants into groups (including a control) and presenting them with articles manipulated in ways that make specific PMT variables more or less salient (e.g., [34–37]). After exposure to the experimental stimuli, participants complete questionnaires that measure intentions to take preventive measures. The findings generally show support for PMT, but the ways in which the components of the appraisal processes interact differ between studies.

Plotnikoff et al. [38] who interviewed a sample of Canadian adults in a longitudinal study found that motivation increases with severity and response efficacy; self-efficacy also played a more substantial role in enhancing behavioral intentions. In another study, participants who perceived higher vulnerability, response efficacy, and self-efficacy were more strongly motivated to perform recommended behaviors. In all cases, motivation and intention to act increased.

Others have applied PMT to analyze the results of surveys that asked people with health conditions to assess the four PMT variables. Plotnikoff et al. [38] found self-efficacy to be the strongest predictor of physical activity and behavioral intention. Unlike experimental studies, survey results indicate that threat appraisals (severity and vulnerability) had no significant effect on intention to perform physical activities. On the other hand, Tulloch et al. [39] observed that self-efficacy, response efficacy, and perceived severity predicted intention to exercise, which, in turn, predicted behavior. However, survey data support the appraisal processes' ability to forecast behavior only for the short term.

Most experimental studies suggest that threat appraisal works with coping appraisal to heighten intention to perform healthy behaviors. For example, Courneya and Hellsten [34] found that individuals who thought of a condition as more severe were more motivated to take action compared to those who saw it as not very severe despite perceived high response efficacy. Survey data, however, show mixed results. Plotnikoff et al. [38] found that coping appraisals influence intention. Tulloch et al. [39], on the other hand, observed both coping and threat appraisals as having an influence on intention and, subsequently, behavior. Another study reports that threat appraisal is most influential in changing behavioral intentions, but self-efficacy, a part of coping appraisal, influences the adoption of prescribed behavior more [40].

Following the propositions of OPM, this study poses the following hypotheses.

 H1: exposure to obesity media content leads to stronger intentions to exercise and maintain a healthy diet.
 H2: attention to obesity media content leads to stronger intentions to exercise and maintain a healthy diet.
 H3: greater exposure to media obesity content leads to greater threat appraisal.
 H4: higher attention to media obesity content enhances coping appraisal.
 H5: people who perceive greater severity and vulnerability (threat appraisal) will show greater intention to exercise and/or maintain a healthy diet.
 H6: people who perceive greater self-efficacy and response efficacy (coping appraisal) will show greater intention to exercise and/or maintain a healthy diet.

## 3. Method

Most studies that examine media effects on knowledge, attitude, and behavior related to health issues have been conducted in urban settings. Iowa was chosen as the study's locale to determine the impact of mediated antiobesity content on audiences in an agricultural state—a food basket rather than a food desert. Still, Iowa ranked 18th on the CDC's [12] list of most obese states, with 29% of residents reporting that they are obese. This figure rose to 31.3% in 2013. A study commissioned by the Robert Wood Johnson Foundation estimated that more than half (54%) of Iowa's population could be obese by 2030 if the trend continues [41].

To arrive at the sample, an online questionnaire was emailed to a random sample of 16,000 student residents of Iowa. The sampling frame was secured from the Office of the Registrar of a Midwestern University. Data gathering was conducted over a seven-week period, beginning January 8, 2013. A total of 633 students returned their completed questionnaire for a response rate of 4%. To diversify the sample, a link to the online survey was posted on the social networking site Facebook, asking the participation of adult Iowa residents. Following this online solicitation, 89 completed questionnaires were received (*N* = 722).

### 3.1. Variables and Their Measure

Exposure to obesity media content pertains to any contact with information about combating, reducing, and preventing obesity found in the mass media. It does not include reports about the growing problem of obesity in the country. Respondents were asked to indicate how often they encounter obesity and related information in each of the following channels: television, radio, print and online newspapers, print and online magazines and journals, and other online sources using a five-point Likert scale (1 = hardly ever; 5 = every day). The responses were averaged to measure exposure to obesity media content.

Attention to obesity media content refers to the level of cognitive consideration respondents give to obesity and related content gleaned from various media channels (TV, radio, print and online newspapers, print and online magazines, and other online sources). For each channel listed, respondents were asked to indicate about how much attention they pay to obesity content using Likert scale items (1 = no attention at all; 5 = pay very close attention).

Perceived severity and perceived vulnerability together form a person's threat appraisal [34]. Perceived severity is defined as one's perception of how serious the consequences of the health issue are. Perceived severity thus refers to the negative consequences an individual associates with obesity and its outcomes. It was measured by using a modified scale developed by the National Cancer Institute (n.d.) [42] to capture people's perception of obesity as a health problem. This index asked respondents to indicate their level of agreement (1 = definitely disagree; 5 = definitely agree) with the following statements: (1) the thought of being obese scares me; (2) when I think about being obese, I feel queasy; (3) if I become obese, my job/career would be endangered; (4) being obese would endanger my personal relationships; (5) how I feel about myself would change if I become obese; (6) I am afraid to even think about obesity; (7) my financial security would be in jeopardy if I become obese; and (8) the health problems I would experience from being obese would last a long time. The National Cancer Institute reports a Cronbach's *α* of 0.78 for this scale; test-retest reliability across an interval of two weeks was 0.76. Perceived severity was computed by averaging the responses to the eight items that comprised the scale.

Perceived vulnerability is one's perception of the likelihood of contracting or developing a health condition [34]. It was assessed using items taken from the scale developed by Plotnikoff and Higginbotham (1988, [43]) who report a Cronbach's *α* of 0.85. The four items used in this study were as follows: if I do not get enough physical activity, I will be at risk (1) for serious health problems, (2) of becoming overweight or obese, (3) for heart disease, and (4) for diabetes. Matching items were used to assess perceived vulnerability without physical activity and a healthy diet with response options ranging from 1 (definitely do not agree) to 5 (definitely agree). Perceived vulnerability was computed by averaging the responses to the four items.

To measure threat appraisal, perceived severity and perceived vulnerability were averaged.

Self-efficacy and response efficacy are the two constructs that make up one's coping appraisal. Self-efficacy or people's beliefs in their ability to perform the recommended response actions were measured using six items from Plotnikoff and Higginbotham [43] who found the index to be internally consistent (Cronbach's *α* = 0.91). The items included are as follows: I can get adequate exercise even when (1) I have many demands at work or at home, (2) I feel depressed, (3) I exercise alone, (4) I get bored with the activities, (5) I do not notice an improvement in my fitness, and (6) I feel tired. The answers to these items (1 = definitely disagree; 5 = definitely agree) were averaged. Parallel scales were used to measure one's self-efficacy in maintaining a healthy diet; however, from the list of questions intended to measure intention to diet items, scales three and four were excluded.

Response efficacy is the perceived effectiveness of the suggested action or behavior in treating or combating the health problem. It also was measured using items from Plotnikoff and Higginbotham [43] who found the index internally consistent (Cronbach's *α* = 0.80). Four items were modified to specifically reference the benefits of exercising and healthy eating. Again, the same eight items were used twice to assess response efficacy for physical activity and maintaining a healthy diet. The eight items were as follows: physical activity or maintaining a healthy diet will or could (1) keep me healthy, (2) reduce my chances of getting serious health problems, (3) reduce my chances of becoming overweight or obese, (4) help me either remain fit or get fit, (5) give me a heart attack, (6) cause muscle and bone injuries, (7) improve my chances of living longer, and (8) improve my overall alertness and thinking. The answers to these items ranged from 1 (definitely disagree) to 5 (definitely agree). The negatively framed items were reverse-coded. The responses were averaged to arrive at a measure of response efficacy.

To measure coping appraisal (for exercising and for maintaining a healthy diet), self-efficacy and response efficacy responses were averaged.

Intention to exercise refers to the degree to which people plan to do physical activities known to reduce and prevent obesity. Behavioral intention is considered the most proximate predictor of behavior [39, 44]. It has been found to have high predictive validity in relation to behavior, indicating that people tend to accurately rate their intention to perform the behavior in question. Meta-analyses findings show 19% to 38% of variance in behavior explained by behavioral intention [45, 46]. To assess intention to exercise, a scale made up of two items developed and validated by Courneya and McAuley [47] was used. Respondents were asked to indicate the degree to which they agree (1 = completely disagree; 7 = completely agree) with the following statements: (1) I intend to exercise regularly over the next month and (2) I intend to exercise regularly over the next six months. These items showed high internal consistency (Cronbach's *α* = 0.91). The answers were averaged to measure intention to exercise.

Intention to maintain a healthy diet was measured by asking respondents to indicate the extent of their agreement (1 = completely disagree; 7 = completely agree) with the following statements: (1) I intend to maintain a healthy diet over the next month and (2) I intend to maintain a healthy diet over the next six months. The answers also were averaged.

## 4. Results

A total of 722 respondents returned their completed questionnaire. The majority (72.6%) were females and European American/Caucasian (89.6%). The respondents' ages ranged from 18 to 83 (*M* = 24.16), with the largest group (78.2%) composed of those between the ages of 18 and 24. A large majority (66.2%) has had some college education. Annual incomes in 2012 ranged from less than $25,000 to more than $65,000 (mode = $25,000).

On average, the respondents had BMIs of 24.9953, considered normal although only 0.0047 away from being overweight (BMI = 25.0). Based on self-reports, 16.3% of the respondents can be classified as obese, while 21.7% were overweight.

The sample reports spending plenty of time online (*M* = 4.53 hours); a few hours were devoted to reading newspapers (*M* = 0.758) or magazines (*M* = 0.301), watching television (*M* = 1.93), and listening to the radio (*M* = 1.35). From these channels, respondents say they occasionally encounter obesity and related information.

### 4.1. Exposure and Attention to Obesity Media Content

To what extent were respondents exposed to obesity media content? On average, respondents encountered this type of information not very often. They reported occasionally seeing obesity discussed or portrayed mostly on television; the least cited sources were magazines and journals. The overall average was 2.89 hours, just over half the time they dedicate to all media channels on a daily basis. When using most of them, they say they devote “more than half” of their attention to obesity-related topics.

Overall, the respondents considered obesity a severe condition (*M* = 3.63; SD = 0.80) to which they see themselves as highly vulnerable (*M* = 4.12; SD = 0.78). They generally thought they were capable of performing actions to combat obesity (*M* = 3.34; SD = 0.80), although this assessment scored the lowest of all the PMT constructs. Response efficacy, the degree to which the recommended behaviors were seen as feasible solutions to the obesity problem, was high (*M* = 4.22; SD = 0.45). Taken together, the respondents saw obesity as a serious threat (*M* = 3.87; SD = 0.65), but they considered the coping mechanisms available to them (exercising and healthy eating) to be effective in offsetting the threat (*M* = 3.78; SD = 0.50). In short, their appraisal of the threat was greater than their appraisal of the efficacy of coping strategies.

Respondents were asked about two behavioral intentions: their intention to exercise and their intention to maintain a healthy diet on the short and longer term (one and six months, resp.). The mean values for these two variables were close, suggesting more than middle-level intention to exercise (*M* = 5.54; SD = 1.49) and maintain a healthy diet (*M* = 5.61; SD = 1.26).

### 4.2. Exposure, Attention, and Behavioral Intentions

H1 posits that exposure to obesity media content heightens one's intention to exercise and maintain a healthy diet. The results of Pearson correlation tests show no significant association between exposure and intention to exercise (*r* = 0.002, *P* = 0.971) and between exposure and intention to eat healthily (*r* = 0.048, *P* = 0.276). Thus, H1 was not supported.

H2 proposes that attention to obesity media content is associated with intention to exercise and maintain a healthy diet. Correlation test results indicate that attention correlated weakly, but significantly, with intention to exercise (*r* = 0.271, *P* = 0.015). However, attention's relationship to intention to maintain a healthy diet was not significant (*r* = 0.032, *P* = 0.779). The results of a simple regression test show that attention was a significant determinant of intention to exercise [*F*(1, 78) = 6.173; *P* = 0.015], contributing 7.3% of the variance. Thus, H2 was only partially supported. That is, attention had a significant effect on intention to exercise but not on intention to maintain a healthy diet.

### 4.3. Exposure and Threat Appraisal

H3 tests whether greater exposure to media obesity content leads to greater threat appraisal, a construct computed by taking the average of responses to perceived severity and perceived vulnerability. Pearson correlation results indicate no significant correlation between the two (*r* = 0.060, *P* = 0.201). Thus, H3 was not supported.

### 4.4. Attention and Coping Appraisal

H4 poses that more attention paid to media obesity content results in greater coping appraisal (for exercising and for healthy eating). Its two components, self-efficacy and response efficacy, were averaged to determine coping appraisal for exercising and for maintaining a healthy diet. The results of two simple regression tests indicate that attention to media leads to greater coping appraisal for exercise [*F*(1, 67) = 4.327, *P* = 0.041] and maintaining a healthy diet [*F*(1, 71) = 5.519, *P* = 0.022]. Thus, H4 was supported. That is, those who paid more attention to the media were more likely to perceive the recommended behaviors as viable responses to combat obesity. The more people paid attention to the media, the more they felt competent to perform the two recommended actions.

### 4.5. Threat Appraisal and Behavioral Intentions

H5 proposes that individuals who perceive greater problem severity and vulnerability to obesity (combined to form threat appraisal) will show greater intention to exercise and/or maintain a healthy diet. The findings of two separate simple regression tests show that threat appraisal is a significant predictor of intention to exercise [*F*(1, 630) = 37.519; *P* = 0.000] and intention to maintain a healthy diet [*F*(1, 628) = 41.360; *P* = 0.000]. Thus, H5 was supported. Those who perceived obesity to be a threatening condition and who also saw themselves to be vulnerable to it showed greater intention to perform the two recommended healthy practices.

### 4.6. Coping Appraisal and Behavioral Intentions

H6 posits that those who perceive greater self-efficacy and response efficacy (which, in combination, measures coping appraisal) will show greater intention to exercise and/or maintain a healthy diet. The results of two separate simple regression tests indicate that coping appraisal for exercising was a significant determinant of intention to exercise [*F*(1, 639) = 304.314; *P* = 0.000]. Coping appraisal for maintaining a healthy diet also was a significant predictor of intention to maintain a healthy diet [*F*(1, 658) = 62.534; *P* = 0.000]. Coping appraisal accounted for 32.3% and 8.5% of the variance in intention to exercise and intention to maintain a healthy diet, respectively. H6 was thus supported. This suggests that evaluating the two practices as effective solutions to obesity as well as seeing one's self as capable of performing them strengthened people's intention to abide by the recommendations.  Table 1 summarizes the results of the simple regression tests.

### 4.7. Cognitive Appraisals and Behavioral Intentions

The results of the previous regression tests show that, as separate variables, threat appraisal and coping appraisal were significant predictors of intention to exercise and intention to diet, but do the two appraisals work together to influence behavioral intentions? Additional analysis was conducted to answer this question. Two multiple regression tests were performed to determine the effect of threat appraisal and coping appraisal (exercise) on intention to exercise and the influence of threat appraisal and coping appraisal (diet) on intention to maintain a healthy diet. The results show that only coping appraisal (exercise) was a significant predictor of intention to exercise [*F*(2, 572) = 138.84, *P* = 0.000]. However, both variables were found to be significant predictors of intention to maintain a healthy diet [*F*(2, 587) = 33.09, *P* = 0.000]. The results suggest that people associate the coping mechanism of maintaining a healthy diet more highly (compared to that of exercising) with combatting obesity. The absence of influence of threat appraisal on intention to exercise suggests that people think of exercising as an activity that offsets health threats besides obesity; thus, they still intend to exercise despite low perceived obesity threat. The results of the multiple regression tests are summarized in Table 2.

## 5. Conclusions

Existing literature on the dynamics of behavioral change related to establishing, promoting, and sustaining healthy lifestyles has so far paid erratic attention to the role of communication, especially the messages people glean from the mass media, in health behaviors. Despite the abundance of antiobesity messages in most media channels, the results of the current study suggest that exposure to such content alone does not significantly affect audience members' intention to practice recommended behaviors. Instead, people must pay close attention to obesity and related information in order for such information to influence behavioral intentions. In this case, attention to mediated health information specifically influenced people's intention to exercise. The finding lends support to the need to distinguish between attention paid to various media messages and frequency of exposure to these messages.

Attention also figures prominently in the two cognitive processes that constitute protection motivation—threat appraisal and coping appraisal [34]. The results of the present study indicate that exposure to mediated health information does not heighten a person's evaluation that obesity poses considerable threat (perceived severity and vulnerability). Instead, attention to media content positively correlated with people's coping appraisals for exercising and maintaining a healthy diet as prescriptions to ward off obesity—their perception of the effectiveness of these recommended behaviors as well as their assessment of their own abilities to perform these behaviors. This lack of association between media exposure and threat appraisal may indicate fatigue with a long-running stream of messages about obesity and the benefits of exercising and healthy eating. These themes have long been woven into a host of media fare, from hard news to entertainment, so much so that constant exposure may have already produced some kind of a ceiling effect.

Considered separately, threat appraisal and coping appraisal significantly influenced intention to exercise and maintain a healthy diet. Taken together, they were found to be significant predictors of intention to diet; only coping appraisal proved to be a significant antecedent of intention to exercise. In all instances, however, coping appraisal was found to be more strongly correlated with behavioral intentions, indicating that self-efficacy and response efficacy are stronger predictors of behavioral intent than perceived severity and perceived vulnerability. Specifically, coping appraisals alone strongly predicted one's intention to exercise and weakly correlated with intention to maintain a healthy diet. This implies that although threat appraisal does influence one's behavioral intent, coping appraisal plays a larger role in strengthening one's intention to exercise and maintain a healthy diet. The finding agrees with those of Plotnikoff et al. [38] who observed self-efficacy as a salient predictor of intention and behavioral outcomes and thus may serve as a potent guide in the design of physical activity interventions for the general population.

The results are in agreement with those of Milne et al. [48] who also found coping appraisal to be a stronger predictor of behavioral intentions than threat appraisal. In contrast, the survey results of Tulloch et al. [39] suggest that threat and coping appraisals work together to influence behavioral intention. Other experiments also found that threat and coping appraisals influenced action intentions (e.g., [34, 35, 38, 49]), although the interactions among the four variables differed.

Other scholars have observed strong behavioral intentions resulting only from self-efficacy. Plotnikoff et al. [38], for example, found no significant influence of threat appraisals. Similarly, Baranowski et al. [50] reported self-efficacy to be the main predictor of practice, but only in combination with response efficacy. In both surveys, however, threat appraisal was not observed to be a significant predictor, unlike in the current study.

The present study extends the tenets of OPM by adding exposure and attention to mass media—through which people receive information regarding obesity and the ways to prevent this condition—into the analytical framework. The results show that attention paid to mediated health information is a significant predictor of coping appraisal as well as behavioral intent. This means that the amount of attention people pay to obesity-related media content enables them to assess their personal ability to perform the recommended practices and thus cope better with the perceived threat. Attention devoted to the mass media as a source of health information also was found to lead to greater motivations to follow practices recommended to offset obesity. In other words, mere exposure to information is not enough. Audience members must be actively involved for subsequent behavioral decision-making.

The findings of the current study buttress those of previous investigations that found both threat and coping appraisals as significant predictors of behavioral intent, offering more empirical evidence for the robustness of OPM. However, the results of the present study differ somewhat in that it found coping appraisal (compared to threat appraisal) a stronger predictor of intention to exercise and maintain a healthy diet. For health communicators, this suggests that even if perceptions of threat are high, people can still be convinced to abide by suggested courses of action by heightening their sense that these actions are viable and within their capabilities and means to perform. When people perceive the recommended response to be effective and actionable, they are more likely to perform the recommended actions despite perceived threats. Thus, the findings also are in agreement with the axioms of the health belief model, which posits that self-efficacy leads to behavior change [50]. The results also conform with the theory of planned behavior, which holds that perceived behavior control (self-efficacy) largely determines intention [44].

The finding that media exposure did not correlate with intention to exercise or intention to maintain a healthy diet suggests that simply disseminating exercise and healthy eating information through the media is not enough to help curb obesity. Rather, these mediated health messages must be presented in ways that grab and hold audience attention. This finding suggests that health communication practitioners should present information that do not stigmatize but motivate people instead. Campaign messages should have “hooks” that can hold audiences' attention long enough to be able to deliver the motivating message. Messages that heighten self-efficacy and response efficacy should be made more salient so that they are the first items people remember upon exposure to obesity and related messages.

Obesity, however, is a complex problem that requires a comprehensive and multifaceted approach of which education is but a component. Indeed, the CDC has suggested a six-pronged attack as part of its “all-hands-on-deck strategy” to combat obesity. In its Weight of the Nation meeting, it argues that (1) people must have access to safe places to exercise; (2) fast-food and chain restaurant menus must be revised to encourage healthier food and beverage options, making them routine; (3) businesses, governments, and others must adopt policies that reduce sugar consumption; (4) the food, beverage, restaurant, and media industries must improve messages about physical activity and nutrition; and (5) health care providers, insurers, and employers must have expanded roles in obesity prevention [51].

What are the best channels for nonstigmatizing, motivational obesity information? The majority of the respondents reportedly peruse online sources and watch television most frequently for health information. These findings point to the best channels to exploit in order to reach the biggest number of people with persuasive reasons to exercise and eat a balanced diet. It should be noted, however, that this recommendation stems from the media habits of a college-age sample.

Because both threat and coping appraisals significantly influenced intention to exercise and maintain a healthy diet, medical practitioners are well advised to offer their patients information that can aid in reasonably appraising the threat and in offering ways that will strengthen coping abilities. Based on the findings, rather than stressing adverse consequences (severity) and risk factors (vulnerability), medical professionals should emphasize the benefits of performing healthy behaviors (response efficacy) as well as the patients' abilities to perform such actions (self-efficacy). Doing so will likely increase coping appraisal and subsequent behavioral intentions.

Although the study expands the roster of variables that may have a bearing on protection motivation, it has four limitations. First, a probability sample would have offered more generalizable results. Second, the online survey precluded the participation of those without computers or Internet access. A mail survey would have reduced these sampling biases and enhanced generalizability. Third, the questionnaire was sent only to those residing in Iowa, a primarily agricultural and relatively racially homogeneous state. Results may differ with a more urban sample. Fourth, the present study considers intentions only at one point in time. A longitudinal design would be able to track changes in behavioral intention over time.

## Figures and Tables

**Table 1:** Summary of simple regression results.

Independent variable	Dependent variable	*R* ^2^	Sig.	*B*	SE *B*	*β*
Attention	Intention to exercise	.073	*P* ≤ .05	.089	.036	.271
Attention	Coping appraisal: exercise	.061	*P* ≤ .05	.036	.015	.246
Attention	Coping appraisal: healthy diet	.072	*P* ≤ .05	.028	.012	.269
Threat appraisal	Intention to exercise	.056	*P* ≤ .001	.551	.090	.237
Threat appraisal	Intention to eat healthily	.062	*P* ≤ .001	.490	.076	.249
Coping appraisal	Intention to exercise	.323	*P* ≤ .001	1.540	.088	.568
Coping appraisal	Intention to eat healthily	.087	*P* ≤ .001	.844	.107	.295

**Table 2:** Summary of multiple regression results.

Independent variables	Dependent variable	*R* ^2^	Sig.	*B*	SE *B*	*β*
Threat appraisal	Intention to exercise	.327	*P* ≤ .001	.046	.085	.020
Coping appraisal				1.536	.101	.564

Threat appraisal	Intention to eat healthily	.101	*P* ≤ .001	.287	.086	.146
Coping appraisal				.650	.127	.224
